# CT- and MRI-based volumetry for Chiari-like malformation and syringomyelia in Pomeranians

**DOI:** 10.3389/fvets.2025.1549205

**Published:** 2025-02-26

**Authors:** Koen M. Santifort, Ines Carrera, Paul J. J. Mandigers

**Affiliations:** ^1^IVC Evidensia Small Animal Referral Hospital Arnhem, Neurology, Arnhem, Netherlands; ^2^IVC Evidensia Small Animal Referral Hospital Hart van Brabant, Neurology, Waalwijk, Netherlands; ^3^Expertise Centre of Genetics, Department of Clinical Sciences, Faculty of Veterinary Medicine, Utrecht University, Utrecht, Netherlands; ^4^Vet Oracle Teleradiology, Norfolk, United Kingdom

**Keywords:** CM/SM, volumetry, segmentation, caudal cranial fossa, ventricular system

## Abstract

**Introduction:**

Volumetric studies in relation to CM/SM have not been reported in Pomeranians. In this study, we aim to (1) report the intermodality agreement between CT- and MRI-based volumetric measurements of the skull and cervical spinal canal, and (2) assess for differences and associations between the volumetric measurements and CM/SM status.

**Methods:**

Pomeranians were included that underwent CT and MRI studies during the period of February 2022–June 2024. Frontal sinus volume (FSV), caudal cranial fossa volume (CCFV), rostral and middle cranial fossa volume (RMCFV), caudal cranial fossa parenchymal volume (CCFPV), rostral and middle cranial fossa parenchymal volume (RMCFPV), cerebellar parenchymal volume (CPV), brain stem parenchymal volume (BSPV), ventricular system volume (VSV), and spinal canal volume between C1-C7 (CSCV) were measured. CCFV to RMCFV volume index (VI) and CCFPV to CCFV (CCFPV%) were calculated. Agreement between MRI- and CT-based quantitative measurements was assessed with intraclass correlation coefficients. Inferential statistical tests including logistic regression modeling were performed to assess for associations between variables and CM/SM status.

**Results:**

For all volumetric assessments that were performed on both CT and MRI, agreement was good or excellent. There were significant differences between SM normal and abnormal dogs for body weight as well as all volumetric parameters except for CCFPV% and RMCFV. Multiple logistic regression showed that a smaller CCFV and larger VSV were associated with SM.

**Conclusion:**

Smaller CCFV and larger VSV are associated with the development of SM in Pomeranians and have increased odds of SM.

## Introduction

The pathogenesis of Chiari-like malformation (CM) and syringomyelia (SM) in dogs is incompletely understood. In the last two decades, numerous studies have evaluated clinical features, morphometric characteristics, and hereditary factors associated with these disorders (1–21). Most of these studies involved the ‘poster breed’ for CM/SM, the Cavalier King Charles Spaniel (CKCS), although other breeds including the Affenpinscher, Chihuahua, Griffon Bruxellois, and, more recently, the Pomeranian dog breed has also been included in large studies. Moreover, a great many other breeds as well as crossbreed dogs have been reported to be affected (22). Collectively, this research has contributed to improve our understanding of these disorders in dogs and humans (23).

For the Pomeranian, our research group has published studies concerning the phenotypic characteristics (15), longitudinal magnetic resonance imaging findings (24), craniocervical 2-dimensional (2D) morphometry (16, 17), and manual external skull measurements (25) of affected versus unaffected dogs. However, volumetric studies in relation to CM/SM have not been reported in this breed.

Morphometric studies in other breeds, most prominently the CKCS, have provided significant clues as to the predisposing anatomical characteristics of dogs with CM/SM. One of the most often cited findings is a decreased size or volume of the caudal cranial fossa and/or relatively increased cerebellar parenchymal volume (3–5, 26–30). Additionally, researchers have found differences between dogs with and without CM/SM for (semi-)quantitative assessments of frontal sinus volume, cranial vault volume indices, spinal canal width, and ventricular system volumes (5, 21, 27, 29, 31, 32). A recent study investigating the prevalence of CM/SM in a cohort or small-sized dog breeds revealed that SM also was found in a number of mesocephalic and dolichocephalic dogs (not necessarily associated with CM), consistent with an even more complex pathogenesis for SM (22).

Most morphometric studies in relation to canine CM/SM have been conducted by making use of MRI, some also or only including computed tomography (CT). Comparability between these modalities for volumetric measurements of the canine skull has not been assessed and most volumetric studies have been based on an original CT-based study in normal dogs (33). It will be valuable to know if measurements performed on MRI and CT images yield comparable results.

In this study, we aim to (1) report the intermodality agreement between CT- and MRI-based volumetric measurements of the skull and cervical spinal canal, and (2) assess for differences and associations between the volumetric measurements and CM/SM status.

## Materials and methods

For this study, Pomeranians were included that underwent both CT and MRI studies during the period of February 2022–June 2024. Signalment was recorded (including sex and neuter status, age, and body weight). Exclusion criteria were (1) MRI or CT scans with artifacts or inadequate image quality, preventing accurate assessment or measurement, (2) age < 12 months (considered skeletally immature), and (3) a prior history or diagnosis of central nervous system (CNS) disease other than CM/SM, ventriculomegaly, supracollicular fluid accumulation, findings related to craniocervical junction abnormalities (CJAs) [e.g., atlanto-occipital overlapping (AOO)], dorsal constriction at C1/C2 [atlantoaxial band (AAB)], atlantoaxial instability (AAI, also referred to as atlantoaxial subluxation), or non-structural disorders such as epilepsy or paroxysmal dyskinesia.

MRI and CT studies were performed under general anesthesia (individualized anesthetic protocols) with a high-field MRI scanner (1.5 T Canon Vantage Elan, The Netherlands) and 16-slice CT scanner (Siemens SOMATOM.go, The Netherlands). Dogs were positioned in sternal recumbency on the horizontal surface of the table with the head in a flexible coil (MRI) or a head rest (CT), both resulting in elevation of the head of about 2–3 cm to the table. MRI sequences obtained included sagittal T2W (echo time (TE) 110 ms, repetition time (TR) 2.6 s, 2.5 mm slice thickness, 256 × 320 matrix), sagittal T1W (TE 10 ms, TR 0.5 s, 2.5 mm slices, 256 × 320 matrix), transverse T2W of the brain (TE 115 ms, TR 4.1 s, 3.0 mm slices, 160 × 192 matrix), transverse T2W of the cervical spinal cord (TE 115 ms, TR 4.1 s, 3.0 mm slices, 160 × 192 matrix) and transverse T1W of the cervical spinal cord (TE 10 ms, TR 0.4 s, 3.0 mm slices, 160 × 192 matrix). Transverse slices at the level of the cervical spinal cord were adjusted to center the syrinx, if visible. In dogs without a visible syrinx on sagittal images, transverse images were acquired at the level of C2-C3 vertebrae. CT scans were performed with the following parameters: 130 kVp tube voltage, 220 mAs tube current, 256 × 256 image matrix, 0.6 and 0.8 mm slice thickness, 0.4 and 0.6 mm slice increment, 1.0 s rotation time, a pitch of 0.6, and Hr60f (Siemens) kernel.

### CM/SM classification

One observer (KS) reviewed the MRI studies and performed classifications for CM/SM using criteria as previously reported, classifying as CM/SM normal or abnormal (15–17).

### CT and MRI volumetry

**Figure 1:**
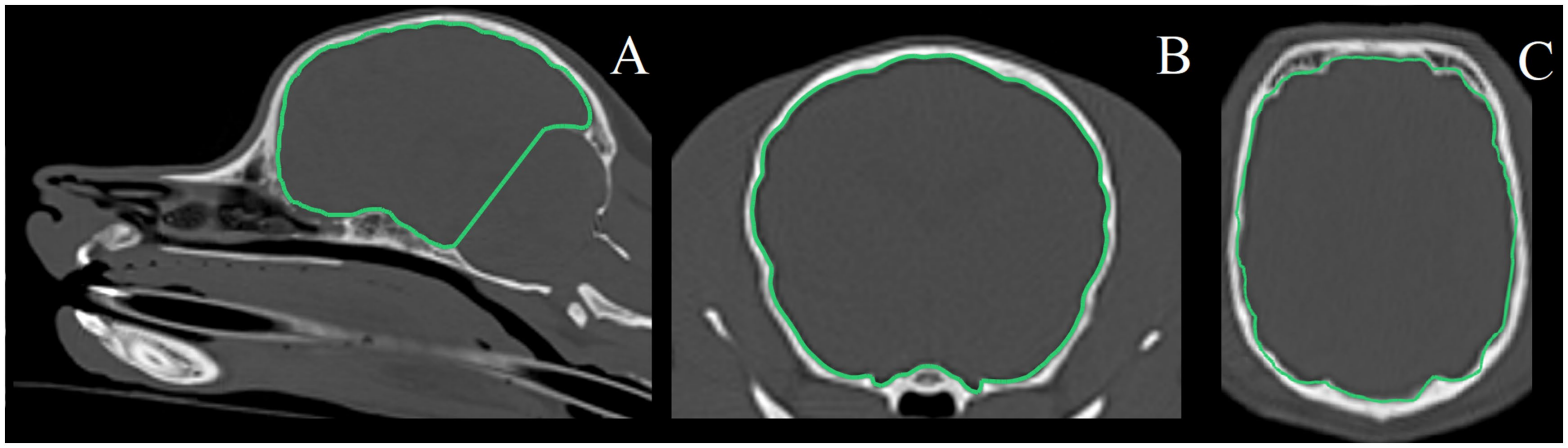
Example of segmentation of volume of interest (rostral and middle cranial fossa volume) on CT images. **(A)** Sagittal plane, **(B)** transverse plane, **(C)** dorsal plane.

One observer (KS) performed quantitative volumetry measurements using dedicated imaging software (Dragonfly 2024 [Computer software]. Comet Technologies Canada Inc., Montreal, Canada; software available at: https://www.theobjects.com/dragonfly). Computer-assisted manual segmentation using the active contouring feature was performed on both MRI and CT images. Window length and width were adjusted as necessary for optimal measurements. Boundaries of volumes of interest were traced on sequential images in three planes of view (transverse plane, and reconstructed dorsal and sagittal planes) (Figure 1).

Tracing was assisted by the software active contouring feature, visually assessed, and manually adjusted where necessary. Values (in mm^3^) were recorded for the following volumes of interest:

(a) Frontal sinus volume (FSV).

**Figure 2:**
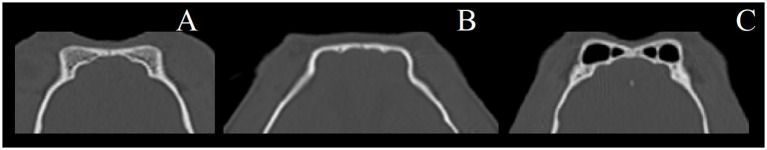
Examples of frontal sinus variations (dorsal plane, CT images). **(A)** Frontal diploë, **(B)** no frontal sinuses, **(C)** frontal sinuses (air-filled).

Frontal sinus boundaries were defined as gas-filled space and/or diploë between the superficial and deep laminae of the frontal bone. CT only (Figure 2).

(b) Caudal cranial fossa volume (CCFV)*.

Caudal cranial fossa boundaries were defined the plane between the tentorium cerebelli osseum and the dorsum sellae rostrally, the skull boundaries laterally, dorsally and ventrally, and the plane between the ventral aspect of the supraoccipital bone and the caudal aspect of the basioccipital bone.

(c) Rostral and middle cranial fossa volume (RMCFV)*.

Rostral and middle cranial fossa boundaries were defined the plane between the tentorium cerebelli osseum and the dorsum sellae caudally and the skull boundaries laterally, dorsally, ventrally, and rostrally.

(d) Caudal cranial fossa parenchymal volume (CCFPV)**.
(e) Rostral and middle cranial fossa parenchymal volume (RMCFPV) **.
(f) Cerebellar parenchymal volume (CPV)**.
(g) Brain stem parenchymal volume (BSPV)**.
(h) Ventricular system volume (VSV).

The ventricular system was defined as to include all ventricles (lateral, third, and fourth).

(i) Spinal canal volume between C1-C7 (CSCV).

The spinal canal boundaries were defined as the plane between the ventral aspect of the supraoccipital bone and the caudal aspect of the basioccipital bone rostrally, the vertebral (spinal) canal itself, and the plane between the caudal aspect of the dorsal lamina of C7 and the floor of the vertebral canal at the level of the C7 caudal endplate. CT only.

* A CCFV to RMCFV volume index (VI) was calculated as VI = CCFV/RMCFV*100.

** Parenchymal volumes were defined as brain tissue present in the respective anatomical compartments or the respective parts of the brain as mentioned.

CCFPV% was calculated as CCFPV/CCFV*100.

**Figure 3:**
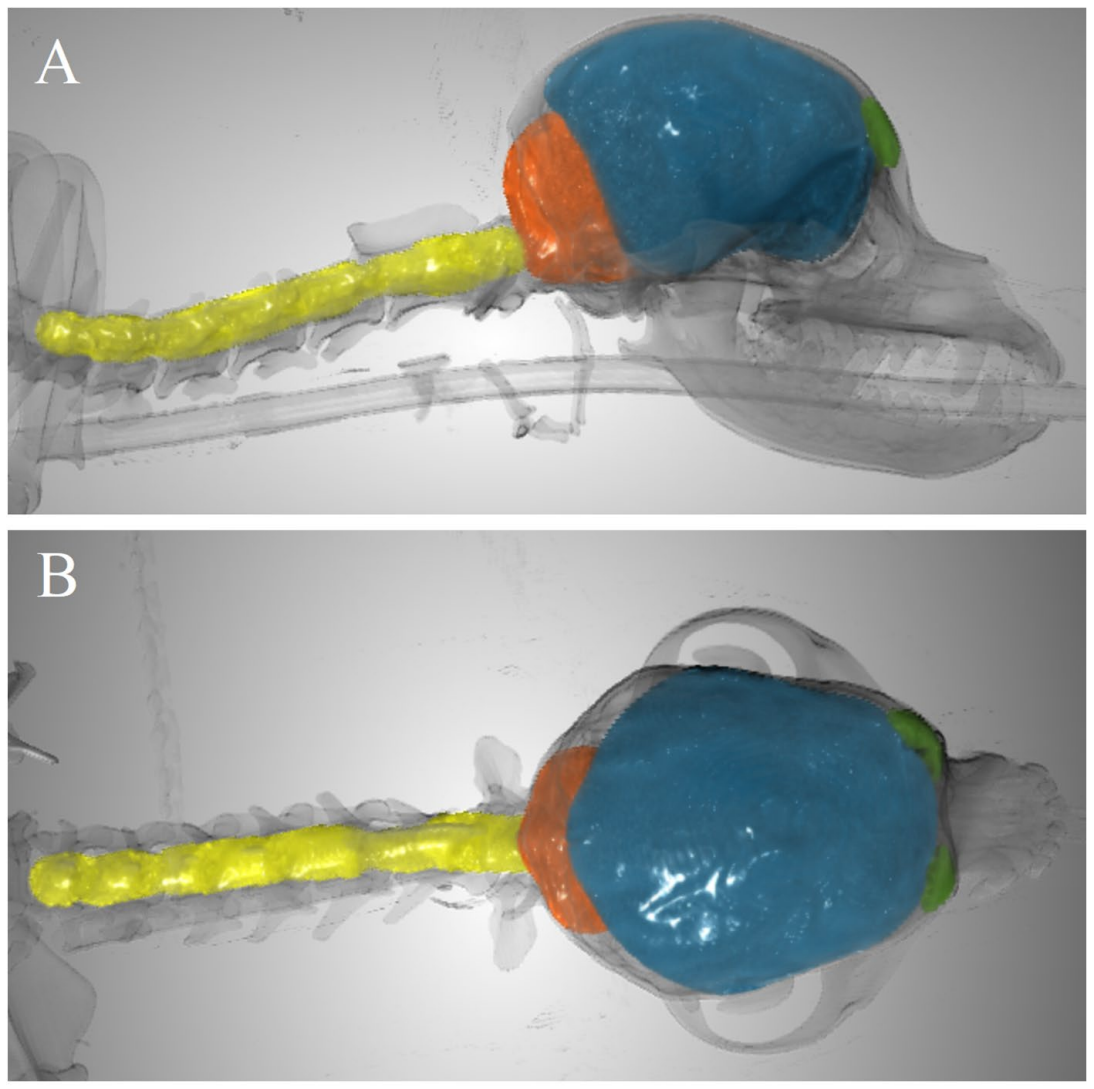
Example of segmented volumes. Frontal sinus volume (green), caudal cranial fossa volume (orange), rostral and middle cranial fossa volume (blue), and spinal canal volume between C1-C7 (yellow) in 3-dimensional image with overlay of the skeleton (transparent). **(A)** Lateral view. **(B)** Dorsal view.

As illustration, Figure 3 includes an example of the volumes segmented for CCFV, RMCFV, and CSCV.

### Statistical analysis

Descriptive statistics are reported using mean (standard deviation) for approximately normally distributed continuous variables and median (interquartile range; IQR) for continuous variables with skewed distributions. Continuous data were tested for normality using a Kolmogorov–Smirnov test. Agreement between MRI- and CT-based quantitative measurements was assessed with intraclass correlation coefficients (ICC, model = two-way random effects, type = agreement, unit = single rating). ICC values including 95% confidence intervals are reported and were interpreted as: < 0.5 = poor; 0.5–0.75 = moderate; 0.75–0.90 = good; > 0.90 = excellent. For further analyses, CT determined values for the volumes of interest were used. Chi-squared tests were used to assess for differences in sex distributions, two-sample t-tests assuming unequal variances (normally distributed data) and Mann Whitney U tests (skewed distributed data) were performed to analyze for differences between CM and SM normal versus abnormal groups of volumetric parameters, age, and body weight. *p*-values of <0.05 were regarded as significant.

Univariable (binary) logistic regression analysis was performed on the datasets with volumetric parameters, age, and body weight as independent (predictor) variables and CM and SM status (normal versus abnormal) as dependent variables. Variables with (Wald) *p*-values of <0.20 on univariable modeling were carried forward to a multiple logistic regression model using manual stepwise regression. Spearman coefficients were calculated between independent variable to assess for collinearity and only one parameters of pairs with coefficients of >0.80 were included in multiple logistic regression modeling. Variables with a (Wald) p-value of <0.05 were regarded as significant. Odds ratios and 95% confidence intervals are given for variables included in the final model. Statistical analyses were performed using Microsoft Excel® v2404 and R v4.3.1.

## Results

### Study population

A total of 137 dogs were included in the study. Median weight (3.2 kg), age (3.0 years) and sex distribution (approximately equal) are presented in Table 1. The prevalences of CM and SM in the study population were 65 and 44%, respectively (Table 2).

**Table 1:** Characteristics of the study population.

Total study population	137 (100%)
Sex	
Female	64 (47%) 53 intact, 11 neutered
Male	73 (53%) 65 intact, 8 neutered
Age (years, IQR)	3.0 years (2.1–4.0)
Weight (kg, IQR)	3.2 kg (2.7–4.0)

IQR, interquartile range.

**Table 2:** Contingency table including numbers and percentages (of total) of included dogs’ CM and SM classifications.

Classification	SM normal	SM abnormal	Total
CM normal	38 (28%)	9 (7%)	47 (35%)
CM abnormal	39 (28%)	51 (37%)	90 (65%)
Total	77 (56%)	60 (44%)	137 (100%)

CM, Chiari-like malformation; SM, syringomyelia.

### Intermodality agreement between CT- and MRI volumetry

**Figure 4:**
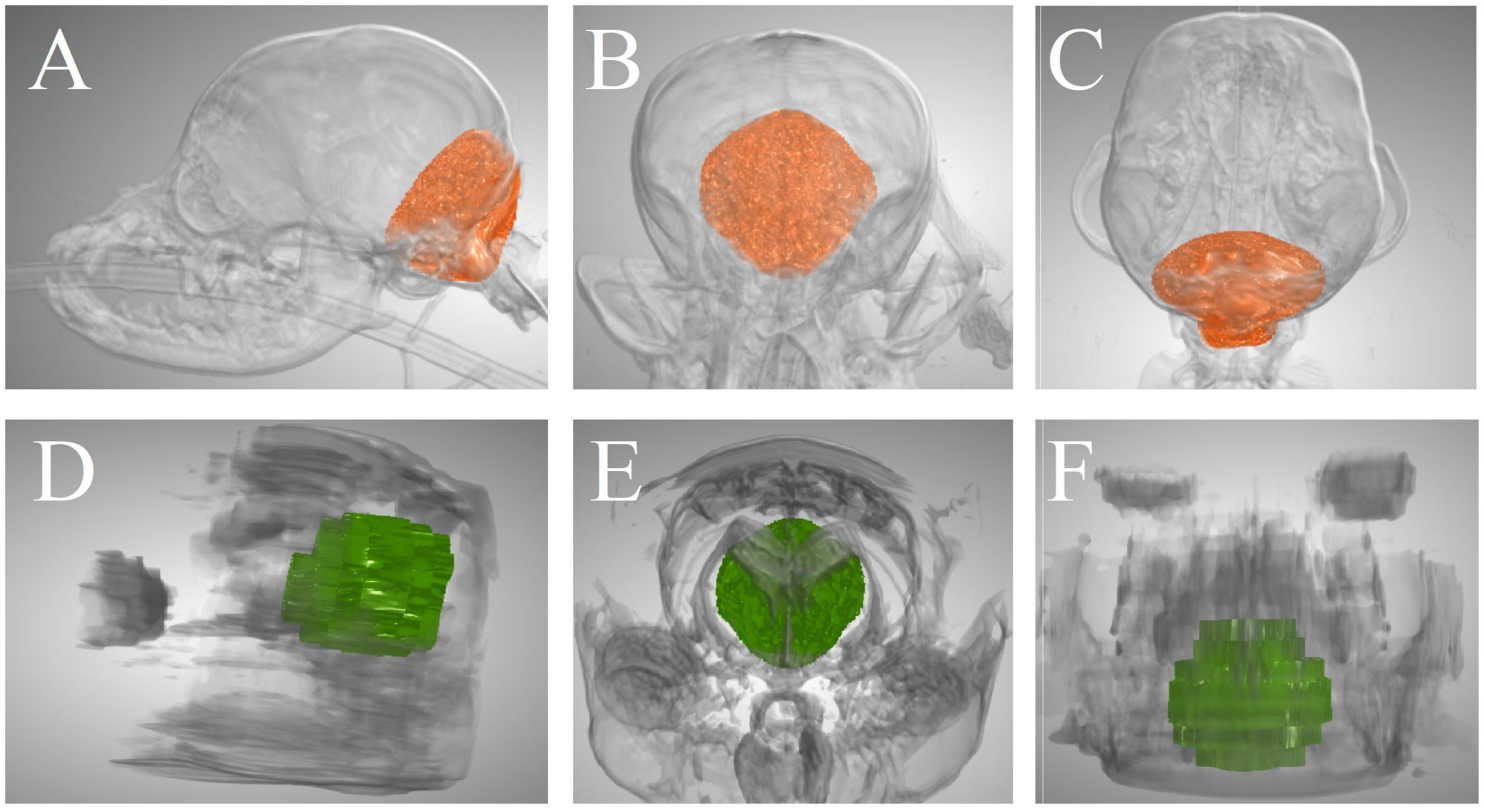
Caudal cranial fossa volume of a Pomeranian dog segmented on CT and MRI. **(A–C)** 3-dimensional CT-based images in lateral view, rostrocaudal view, and dorsal view, respectively. **(D–F)** 3-dimensional MRI-based images in lateral view, rostrocaudal view, and dorsal view, respectively. The CT-based volume was 7,805 mm^3^ and the MRI-based volume was 9,101 mm^3^.

For all volumetric assessments that were performed on both CT and MRI, ICCs were good (CCFV, RMCFV, RMCFPV, CPV, and BSPV) or excellent (VSV) (Table 3). Of note, 95% confidence interval lower limits were poor (RMCFV, and CPV) or moderate (CCFV, CCFPV, RMCFPV, and BSPV) for all ICCs. Figure 4 illustrates the CCFV of a dog based on CT and MRI.

**Table 3:** Intermodality agreement between CT- and MRI volumetry.

Parameter	ICC	95% CI
CCFV	0.75	0.54–0.86
RMCFV	0.75	0.16–0.90
CCFPV	0.81	0.55–0.90
RMCFPV	0.87	0.69–0.94
CPV	0.89	0.45–0.96
BSPV	0.81	0.71–0.87
VSV	0.94	0.37–0.98

BSPV, brain stem parenchymal volume; CCFPV, caudal cranial fossa parenchymal volume; CCFV, caudal cranial fossa volume; CI, confidence interval; CPV, cerebellar parenchymal volume; CSCV, spinal canal volume between C1-C7; ICC, intraclass correlation coefficient; RMCFPV, rostral and middle cranial fossa parenchymal volume; RMCFV, rostral and middle cranial fossa volume; VSV, ventricular system volume.

### Volumetry

**Figure 5:**
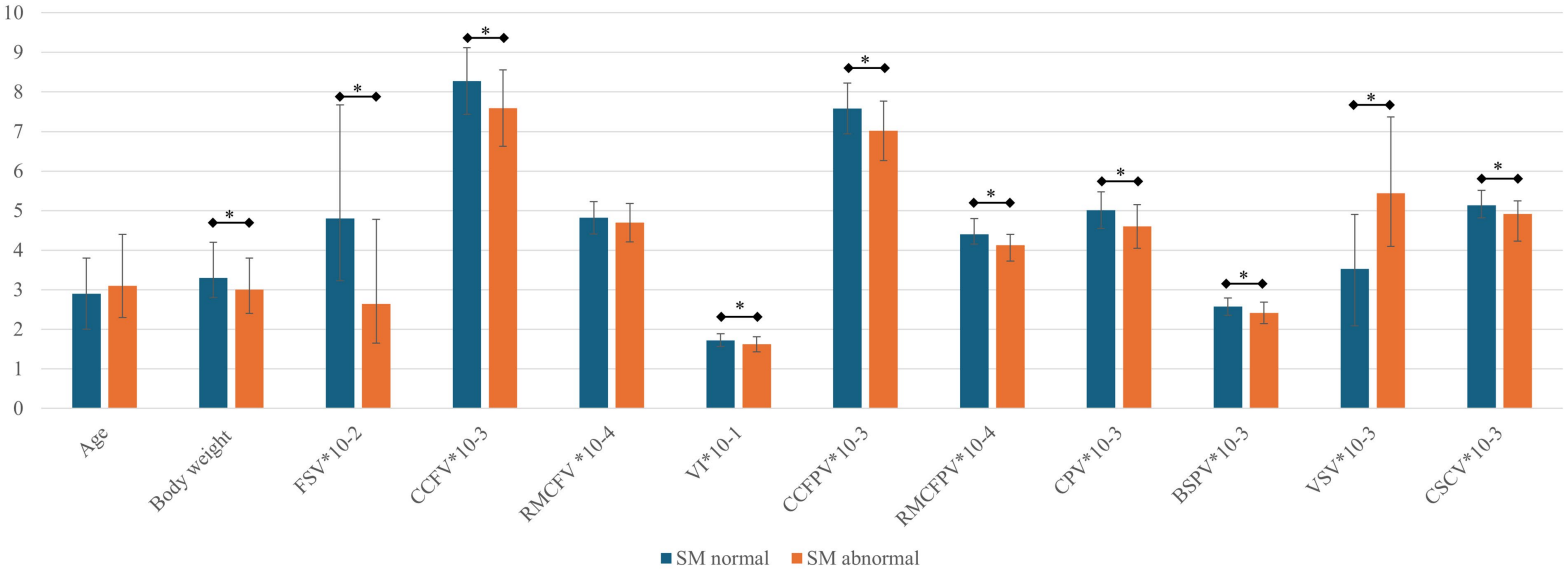
Bar graph for parameters between SM normal and abnormal dogs. The mean or median (error bars indicating standard deviation or interquartile range respectively) are depicted for each parameter (see Table 4). BSPV, brain stem parenchymal volume; CCFPV, caudal cranial fossa parenchymal volume; CCFV, caudal cranial fossa volume; CPV, cerebellar parenchymal volume; CSCV, spinal canal volume between C1-C7; RMCFPV, rostral and middle cranial fossa parenchymal volume; RMCFV, rostral and middle cranial fossa volume; SM, syringomyelia; VI, volume index; VSV, ventricular system volume. All presented values except for age (years), body weight (kg), and VI (%) are in mm^3^. *, *p* < 0.05. Parameters are numerically adjusted to fit the y-axis (10^-x^).

Table 4 includes mean and median values for CT-based volumetric parameters as well as results of testing for significant differences between CM/SM normal versus abnormal dogs. These data are graphically depicted in Figure 5.

**Table 4:** CT-based volumetric parameters for the study population of 137 dogs and per CM/SM status.

Parameter	Study population	CM normal	CM abnormal	*p*-value	SM normal	SM abnormal	*p*-value
Sex	73/64	27/20	46/44	0.06	41/36	32/28	1.00
Age	3.0 (2.1–4.0)	2.9 (2.0–3.8)	3.1 (2.2–4.2)	0.41	2.9 (2.0–3.8)	3.1 (2.3–4.4)	0.26
Body weight	3.2 (2.7–4.0)	3.3 (2.6–4.0)	3.2 (2.7–4.0)	0.85	3.3 (2.8–4.2)	3.0 (2.4–3.8)	**<0.01**
FSV	404 (196–635)	449 (186–647)	403 (200–630)	0.73	480 (323–767)	264 (165–478)	**<0.01**
CCFV	8,033 (910)	8,211 (830)	7,940 (939)	0.09	8,318 (810)	7,668 (906)	**<0.01**
RMCFV	47,639 (4,459)	48,385 (4,327)	47,249 (4,501)	0.15	48,175 (4,046)	46,951 (4,887)	0.12
VI	16.91 (1.76)	17.02 (1.58)	16.85 (1.86)	0.57	17.31 (1.59)	16.39 (1.85)	**<0.01**
CCFPV	7,064 (773)	7,154 (643)	7,017 (797)	0.29	7,340 (658)	6,710 (770)	**<0.01**
CCFPV%	90.04 (83.03–93.19)	88.34 (82.55–92.04)	90.59 (83.42–93.53)	0.21	90.04 (84.38–92.47)	90.29 (82.21–93.61)	0.95
RMCFPV	42,373 (40,118–45,804)	43,428 (40,455–47,025)	41,938 (39,612–45,689)	0.13	44,073 (41,528–48,011)	41,232 (37,233–43,961)	**<0.01**
CPV	4,831 (542)	4,875 (485)	4,809 (571)	0.48	5,011 (463)	4,601 (552)	**<0.01**
BSPV	2,503 (254)	2,536 (226)	2,486 (267)	0.25	2,572 (223)	2,416 (267)	**<0.01**
VSV	4,425 (2,886–6,120)	4,438 (2,329–6,338)	4,403 (3,196–6,102)	0.84	3,528 (2,084–4,899)	5,443 (4,095–7,317)	**<0.01**
CSCV	5,030 (4,715–5,412)	4,975 (4,712–5,357)	5,046 (4,717–5,488)	0.73	5,133 (4,814–5,516)	4,914 (4,224–5,250)	**<0.01**

BSPV, brain stem parenchymal volume; CCFPV, caudal cranial fossa parenchymal volume; CCFV, caudal cranial fossa volume; CPV, cerebellar parenchymal volume; CSCV, spinal canal volume between C1-C7; FSV, frontal sinus volume; RMCFPV, rostral and middle cranial fossa parenchymal volume; RMCFV, rostral and middle cranial fossa volume; VI, volume index; VSV, ventricular system volume. All presented values except for age (years), body weight (kg), sex (M/F), and VI (%) are in mm^3^. For variables with skewed distributions, medians, interquartile ranges, and *p*-values (Mann Whitney *U* test) are presented and for normally distributed variables, means, standard deviations, and *p*-values (two-sample *t*-tests assuming unequal variances) are presented. Gray shaded cells: non-significant results.

The *p*-values that are <0.05 are in bold = significant - the shading that was present in our submitted version shaded the insignificant results and values.

There were no significant differences between CM normal and abnormal dogs for sex distribution, age, body weight, or any volumetric parameter.

There were significant differences between SM normal and abnormal dogs for body weight, as well as all volumetric parameters except for CCFPV% and RMCFV. There was no difference in sex distribution or age between SM normal versus abnormal groups.

Univariable logistic regression with CM status as dependent variable yielded CCFV (*p* = 0.10), RMCFV (*p* = 0.15), and RMCFPV (*p* = 0.11) as parameters with a *p*-value of <0.20. Multiple logistic regression did not result in any significantly associated parameters.

Upon univariable logistic regression with SM status as dependent variable, all tested independent variables with the exception of sex distribution, age, and CCFPV% had a p-value of <0.20 [BW (*p* = 3.4*10^−3^), FSV (*p* = 3.6*10^−3^), CCFV (*p* = 1.0*10^−4^), RMCFV (*p* = 0.10), VI (*p* = 3.2*10^−3^), CPV (*p* = 2.7*10^−5^), BSPV (*p* = 3.7*10^−4^), CCFPV (*p* = 1.0*10^−7^), RMCFPV (*p* = 1.2*10^−4^), VSV (*p* = 1.0*10^−4^), and CSCV (*p* = 5.1*10^−4^)].

**Figure 6:**
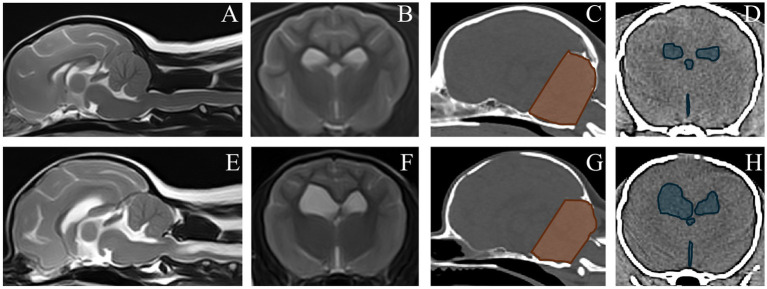
Magnetic resonance images and computed tomography images of a dog without SM (upper row) and with syringomyelia (SM) (bottom row). **(A–E)** MRI, sagittal T2W. **(B,F)** MRI, transverse T2W at the level of the interthalamic adhesion. Segmented caudal cranial fossa volumes (CCFV) and ventricular system volumes (VSV) are shown on corresponding CT images **(C,D,G,H)**. The dog without SM had a CCFV of 8,675 mm^3^ and a VSV of 1703 mm^3^. The dog with SM had a CCFV of 6,817 mm^3^ and a VSV of 7,781 mm^3^.

Multiple logistic regression modeling with SM as dependent variable yielded a best fit model including CCFV (odds ratio for every cm^3^ increase in CCFV = 0.41, 95% confidence interval 0.25–0.68, *p* = 5.1*10^−4^) and VSV (odds ratio for every cm^3^ increase in VSV = 1.40, 95% confidence interval 1.17–1.67, *p* = 2.2*10^−4^); a smaller CCFV and larger VSV were associated with SM. For illustration, Figure 6 includes MRI and CT images of dogs classified as SM normal and abnormal with CCFV and VSV segmentation highlighted.

## Discussion

### CT and MRI intermodality agreement for volumetric parameters

In our study, good or excellent agreement between CT and MRI volumetric measurements was found. Such agreement has not specifically been reported before. Of importance is that the lower limits of the 95% confidence intervals were poor to moderate. This means that it is possible that agreement between these two modalities is indeed poor to moderate, rather than good or excellent. In any case, the agreement was not perfect, implying that CT-based and MRI-based measurements and analysis thereof might result in different outcomes. This impacts the interpretation and comparison of different studies using either modality for volumetric assessments.

Many previous studies using MRI referred back to a CT-based publication for validation of their MRI-based measurements (34). Those studies looking at, for instance, CCFV or associated linear measurements in CKCS in relation to SM have mostly employed MRI-based measurements (3, 4, 7, 8, 26, 29, 32, 35). MRI lacks spatial resolution in comparison to CT and bony landmarks are less clearly defined, hampering precise measurements. Moreover, slice thickness and thus voxel dimensions differ between studies, which subjects comparison between absolute and relative values between studies to yet more variability than other factors such as interobserver variability (16).

Unsurprisingly, linear measurements have been found to be associated with volumetric measurements (34). Linear measurements require less time than volumetry and are easier to perform and possibly use for clinical cases. Many of the cited publications on CM/SM in dogs included 2D, linear measurements. Although linear and volume measurements are associated, differences between linear measurements associated with volume and measured volumes will arise. Simply put, measuring associated, indirect indicators of volumes, is not identically valuable compared to actual measurements of the volume (36). Differences between published study results both in comparison to each other and to our study might be explained by these differences in methods (i.e., linear versus volume measurements and CT versus MRI measurements).

For our study, we have made use of specific software that enabled volumetric measurements as detailed in the materials and methods section. There are numerous other software options available, as listed in the references cited above, that could be considered and there are no studies that evaluate the differences between these options specifically. Considerations for the selection of particular software may include for instance: user-friendliness, licensing costs versus open-access, hardware requirements, and suitability to study designs.

### Chiari-like malformation

We identified no statistically significant differences between dogs with or without CM for any of the studied volumetric variables. While some morphometric differences have been identified between CM affected and non-affected Pomeranians (e.g., foramen magnum height and distance between the dorsal arch of the atlas and the foramen magnum), many other variables studied so far were not significantly different (17, 25). This may be partly explained by questionable reliability of CM grading, with suboptimal interobserver agreement having been identified for both the CKCS and Pomeranian dog breeds (16, 33). Otherwise, the lack of any such differences may represent dissimilarities between Pomeranians and other breeds like the CKCS in relation to the pathogenesis of CM/SM and to what actually constitutes CM in dogs in general; we might not be talking about the exact same thing in all studies as the exact definitions and grading systems are imperfect and partly open to interpretation. Moreover, the effect of positioning for imaging studies is often not accounted for and existing classification schemes reduce CM to a categorical variable with inherent limitations.

Of particular interest might be the lack of associations between CCFV and CM. This is in agreement with the lack of such an association for caudal cranial fossa area (a 2D measurement) as studied previously in Pomeranians (17). This might seem in disagreement with some publication centering on other breeds, like the CKCS (7, 8, 26, 29). However, it must be remembered that these studies looked for differences in CCFV between dogs in relation to SM status, not CM status. As almost all CKCS have CM, these studies were not able to assess for differences between CM affected and unaffected dogs. We too found differences in CCFV as well as VI between dogs with and without SM. This will be discussed below.

### Syringomyelia

For dogs with SM versus without SM, we identified significant differences between Pomeranians with and without SM for numerous volumetric parameters as well as body weight.

#### Body weight

Body weight was lower for the dogs with SM than dogs without SM. Interestingly, body weight was not accounted for as a variable in many studies looking at CM/SM in CKCS; a note of great importance as emphasized by Schmidt et al. (35). An effect of body weight on morphometric comparisons among and between dogs with or without SM exists (35). We expected no different for Pomeranians and already recognized this in a previous study (25). Body weight can partly substitute for ‘size’ but body condition has great implications for body weight as well (e.g., dogs of similar size may differ in body weight due to difference in muscle and fat mass). Like Schmidt et al. (35), we did not include body condition scores and can therefore not correct body weights to more accurately reflect size. Nevertheless, assuming – as is our clinical impression – that most of the included dogs had an average body condition score, the difference in body weight implies that dogs with SM were overall smaller than those without. Like body weight, many of the variables we studied are a related to overall size of the dog [allometric relationships between variables exist (see discussion in Santifort et al. (25))]. It can therefore be concluded that, as far as SM in Pomeranians is considered, size matters.

#### Frontal sinus volume (FSV)

FSV was smaller in dogs with SM than dogs without SM. There is one previous study that looked at frontal sinus morphology in relation to SM in small breed (<15 kg) dogs (31). This study did not measure FSV, but classified dogs into groups based on frontal sinus morphology as ‘absent (no air and no diploë)’, ‘miniscule (some diploë may be seen)’, ‘small (but normal)’, and ‘normal (air-filled)’ (31). The imaging modality used in that study was MRI. The absent and miniscule categorized dogs had higher odds to be affected by SM than dogs in the other categories. In our study, we actually measured FSV on CT images (with better delineation of the bony boundaries) but the conclusion is the same: a smaller frontal sinus is linked to SM. This by no means implies a causal relationship. The association could be coincidental as a smaller FSV may simply be associated to the same factors or be part of the same overall pathogenesis leading to SM. Similarly, Scrivani et al. postulated that SM *‘in many small breed dogs…develops as a result of global malformation of the entire cranial cavity or supratentorial portion of the cavity and is not limited to the infratentorial portion of the cranial cavity’* (31). Just based on FSV, regarding Pomeranians, our results support this theory. However, the argument of allometry needs to be considered (see above under ‘body weight’).

#### Caudal cranial fossa volume (CCFV), rostral and middle cranial fossa volume (RMCFV) and volume index (VI)

CCFV as well as VI were smaller in dogs with SM than dogs without SM, while there was no statistically significant difference for RMCFV. It follows that while the CCFV is both absolutely and relatively smaller in Pomeranians with SM, the RMCFV is not. CCFV was also one of two (the other being VSV) variables included in the multivariable logistic regression model, identifying it as not only significantly different between SM affected and unaffected dogs, but also as robustly associated with SM status in comparison to other studied variables. In our previous study using 2D measurements, caudal cranial fossa area measured on MRI and CT was significantly smaller in dogs with SM as well (17). These findings are in line with some previous studies in other dog breeds (7, 26, 29). However, there are studies that did not identify statistically significant differences (3, 5, 27, 28, 32). Importantly, some studies that actually (directly) measured CCFV did not identify differences between affected and non-affected dogs (3, 32). In a human meta-analysis, posterior cranial fossa volume was associated with SM (36). However, size considerations and impact thereof (see above under ‘body weight’) are less of a confounder in these human studies (35). Hence, future canine studies considering this parameter will provide more information for the continued debate on relevance of CCFV in the pathogenesis of SM.

#### Ventricular system volume (VSV)

VSV was larger in dogs with SM than dogs without SM. VSV was also one of two (the other being CCFV) variables included in the multivariable logistic regression model, identifying it as not only significantly different between SM affected and unaffected dogs, but also as robustly associated with SM status in comparison to other studied variables. The association of SM and VSV has been reported in the CKCS and, indirectly, in Chihuahuas as well (5, 6). No association was found in a study that assessed lateral ventricles parameters in 2D and categorically (i.e., grouped as 0–14% = absent ventriculomegaly; 15–25% = moderate ventriculomegaly; >25% = severe ventriculomegaly) (21). As VSV is a continuous variable and volumetry is more accurately assessed by 3D than 2D measurements, current evidence seems to support a role of VSV increase in dogs with SM. Whether this variable is causative or secondary to, for instance, altered cerebrospinal fluid (CSF) flow dynamics (i.e., merely being associated with SM in a non-causative, coincidental manner) remains to be shown. Longitudinal studies evaluating both VSV as well as SM development might help to gain more insights to begin to answer this question.

#### Other volumetric parameters (CCFPV, CCFPV%, RMCFV, RMCFPV, CPV, BSPV, and CSCV)

While CCFPV was significantly smaller in dogs with SM, CCFPV% was not. This indicates that relative overcrowding of the caudal cranial fossa does not seem to be implicated in the pathogenesis of SM in Pomeranians as has been suggested for the CKCS (4, 5, 29, 37, 38). These studies differed in many aspects to ours and among themselves, such as (1) mainly CKCS dogs included, (2) some of the studies used 2D measurements, (3) weight was not always considered (see discussion under ‘body weight’) for absolute measurements, and (4) ventricular size was not accounted for. The differences in study results could be due to each or a combination of these factors. Likewise, though RMCFPV, CPV, and BSPV were significantly smaller in dogs with SM, these are influenced by overall size of the dog (see discussion under ‘body weight’) and also ventricular dimensions. The Monro-Kellie Doctrine dictates that increase in one intracranial compartment (CSF, blood, parenchyma) has to be compensated by decrease in another; in dogs with increased ventricular volume, brain parenchyma volume (mostly white matter) is reduced (39). Our finding of association between VSV and SM is indicative of influence on the parenchymal volume factors as well. This is supported by the fact that these parenchymal volumes were not valuable for the logistic regression model.

CSCV was smaller for dogs with SM than dogs without SM in our study. Spinal canal width (a 2D measurement) at C2-3 was increased for dogs with SM in another study (27). Differences between study methods could explain this contrast in findings (e.g., dog breeds, positioning for scans, 2D versus 3D). As for the other parameters, CSCV was not of value in the logistic regression and the argument of allometry is of importance to consider (i.e., smaller dogs have smaller CSCV, see discussion above under ‘body weight’). While absolute volume of the spinal canal might be interesting, it is important also to consider the effect of dynamics and its postulated role in the development of SM (40). Cervical spinal cord movements and the effect thereof on volumes measured may have an important effect on SM development. Cervical stabilization has even been proposed as an (adjunctive) surgical method to treat SM in people (40). Pomeranians are often affected by numerous other CJAs that might influence cervical and CSF dynamics. The role of CSCV and dynamics in the development of SM is therefore complex. Future studies assessing associations between CJAs and SM in Pomeranians could help to gain more understanding of their influence.

#### Age and sex

Consistent with the literature, no sex predispositions were identified for CM/SM in dogs in this study. Age was not identified as significantly different between the SM affected and non-affected dogs, like in two previously published studies (17, 25) but unlike our other study with the largest study population (15). In another previous study on a small cohort of Pomeranians, we found that SM was progressive over time (24). Other studies have also identified SM to be associated with age or have characterized SM to be progressive over time in CKCS (28, 41–46). While we did not identify an association with age in this study, SM was not evaluated longitudinally over time. Differences in the studied number of dogs between this and previous studies could also have impacted the lack of a significant difference in this study (15, 17).

### Limitations

There are limitations to this study, including the lack of intra- and interobserver reliability assessments and 3D sequences for MRI studies that would facilitate more accurate volumetry compared to 2D sequences, and, as for other studies, the possibility of misclassification of dogs with CM (16, 33). Nevertheless, our study provides valuable insights into the pathogenesis of CM/SM in Pomeranians.

## Conclusion

In conclusion, smaller CCFV and larger VSV are associated with increased odds of SM in Pomeranians. Such parameters, along with others that we have studied (14, 16, 17, 25) may be taken into account in the overall assessment of CM/SM and the selection of dogs for breeding programs of this breed. The correlation between body weight and SM and its implication on overall size might be considered in breeding practices but further research accounting for body condition scores would be valuable to verify such findings.

## Data Availability

The raw data supporting the conclusions of this article will be made available by the authors, without undue reservation.
